# The Structures of ZnCl_2_-Ethanol Mixtures, a Spectroscopic and Quantum Chemical Calculation Study

**DOI:** 10.3390/molecules26092498

**Published:** 2021-04-25

**Authors:** Payam Kalhor, Yaqian Wang, Zhiwu Yu

**Affiliations:** MOE Key Laboratory of Bioorganic Phosphorous Chemistry and Chemical Biology, Department of Chemistry, Tsinghua University, Beijing 100084, China; kalhor.payam@yahoo.com (P.K.); wangyq19@mails.tsinghua.edu.cn (Y.W.)

**Keywords:** ethanol, ZnCl_2_, excess spectroscopy, two-dimensional correlation spectroscopy, spodium bond

## Abstract

We report in this article the structural properties, spectral behavior and heterogeneity of ZnCl_2_-ethanol (EtOH) mixtures in a wide-composition range (1:3 to 1:14 in molar ratios), using ATR-FTIR spectroscopy and quantum chemical calculations. To improve the resolution of the initial IR spectra, excess spectroscopy and two-dimensional correlation spectroscopy were employed. The transformation process was suggested to be from EtOH trimer and EtOH tetramer to EtOH monomer, EtOH dimer and ZnCl_2_-3EtOH complex upon mixing. The theoretical findings showed that increasing the content of EtOH was accompanied with the flow of negative charge to ZnCl_2_. This led to reinforcement of the Zn**←**O coordination bonds, increase of the ionic character of Zn‒Cl bond and weakening and even dissociation of the Zn‒Cl bond. It was found that in some of the ZnCl_2_-EtOH complexes optimized at the gas phase or under the solvent effect, there existed hydroxyls with a very special interactive array in the form of Cl‒Zn^+^**←**O‒H**^…^**Cl^−^, which incredibly red-shifted to wavenumbers <3000 cm^−1^. This in-depth study shows the physical insights of the respective electrolyte alcoholic solutions, particularly the solvation process of the salt, help to rationalize the reported experimental results, and may shed light on understanding the properties of the deep eutectic solvents formed from ZnCl_2_ and an alcohol.

## 1. Introduction

Aliphatic alcohols are regarded as very important chemicals with a broad range of applications [1,2,3,4]. They are amphiphilic substances, making them miscible with a wide range of polar and nonpolar substances [5]. The amphiphilicity of alcohols results in a high complexity of their structures rendered by various intermolecular interactions, most importantly hydrogen bonds (H-bonds) [6,7,8]. The H-bonded networks in alcohols are readily susceptible to disruption and reformation by chemical perturbations [9,10,11]. Structural properties of alcohols in pure state or mixed with cosolvents and their relationship with H-bonds have been the subject of numerous experimental and computational studies [12,13,14,15,16,17,18,19,20,21]. When it comes to electrolytes, their solvation in alcohols and aqueous alcohol solutions are of great significance in many industrial and natural processes [22,23,24]. So, the mixtures of alcohols with organic or inorganic salts have been vastly investigated, mostly focusing on understanding the physicochemical properties of the systems [22,25,26,27,28,29,30,31,32,33]. Of particular interest, it has been reported that the dissolution of some salts in certain alcohols in appropriate molar ratios leads to a significant drop in freezing points with respect to the composing pure components, forming the neoteric class of solvents coined as deep eutectic solvents (DESs) [23,24,34]. In this regard, the DESs of ZnCl_2_-ethylene glycol (EG) [35], ZnCl_2_-hexanediol [35], choline chloride (ChCl)-methanol [36], ChCl-EG [37] and ChCl-glycerol [38] mixtures have been reported. But, regretfully, little is known about the underlying solubility phenomena, the structural properties and the reasons behind the deviations from the ideal behavior in such alcohol-involving mixtures; additionally, the published data of this type are mostly available for aqueous systems [39,40,41,42,43].

Among different types of alcohols, ethanol (EtOH) in pure state or cosolvent-mixed solutions has been explored to a larger extent to understand the structures and physicochemical properties [44,45,46,47,48,49,50,51,52,53]. But, when it comes to the salt-EtOH/aqueous EtOH solutions, there are only a few studies. It has been proposed that in the dissolution process of inorganic salts in ethanol (EtOH) and its derivative, it is the electrostatic force between the dissociated ions and solvent molecules that governs the solvation [33]. Moreover, alcohols are expected to form solvation structures around the dissociated ions by forming H-bonds [54,55]. Nose et al. [56] studied the effects of several metal halides on the H-bonding properties of EtOH-water solutions using nuclear magnetic resonance (NMR) spectroscopy. They found that the studied acids and phenolic compounds strengthened the EtOH-water structures and enhanced the proton exchange between EtOH and water. They also found that many salts dissociated the structure of water in the EtOH-water mixtures, while a few ones such as MgCl_2_ and KF had strengthening effects. Yamauchi et al. [9] measured the Raman spectra of pure glassy EtOH and its mixtures with LiCl at different concentrations. In the OH region, two peaks at 3309 and 3373 cm^−1^ were resolved, which grew as the concentration of LiCl increased. The peak at 3309 cm^−1^ was attributed to the ν(O−H) in the second solvation shell around the Cl^−^ and the peak at 3373 cm^−1^ was assigned to ν(O−H) directly H-bonded to Cl^−^. Glinski et al. [57] explored the solvation of CeCl_3_ and PrCl_3_ in aqueous and pure EtOH by measuring the conductivities, densities and ultrasonic velocities. They found that lanthanide chlorides are in general weak electrolytes in EtOH. They indicated that as the concentration of the salt increases, two chlorides in the first solvation sphere of the metal cation increase to three.

Despite all of these efforts, there exists a considerable dearth of reliable microscopic insights into the heterogeneity of electrolyte alcoholic solutions. In particular, a clear picture of the structures of the highly applicable organic solvent EtOH under the influence of inorganic salts at a relatively wide range of compositions and identification of the prominent structures are highly needed to advance the knowledge. In addition, characterization of the underlying intermolecular interactions and determining their nature, and quantifying the interatomic charge transfer and its role in the interactions have not been explored sufficiently. These studies become more urgent when knowing that several applicable DESs are formed by combining only salts and pure alcohols [35,36,37,38].

These scarcities encouraged us to undertake the present work where attenuated total reflection Fourier transform infrared (ATR-FTIR) and quantum chemical calculations including Hirshfeld charge analysis have been used to study the spectral behavior and quantum chemical properties of a wide range of compositions of ZnCl_2_-EtOH mixtures. Excess absorption spectroscopy [10,20,58,59] and two-dimensional correlation spectroscopy (2D-COS) [60] were particularly utilized to enhance the resolution of the overlapped initial IR bands. Acquiring this molecular-level knowledge would help qualitatively rationalize the observed macroscopic properties such as vapor pressure of similar mixtures. The knowledge would also advance the current understanding on the behavior of DESs consisting of ZnCl_2_ and an alcohol and helps design novel and more efficient DESs.

## 2. Results and Discussion

### 2.1. ATR-FTIR Analysis of the Interactions between ZnCl_2_ and EtOH in ν(O−H) and ν(C−H) Regions

**Figure 1:**
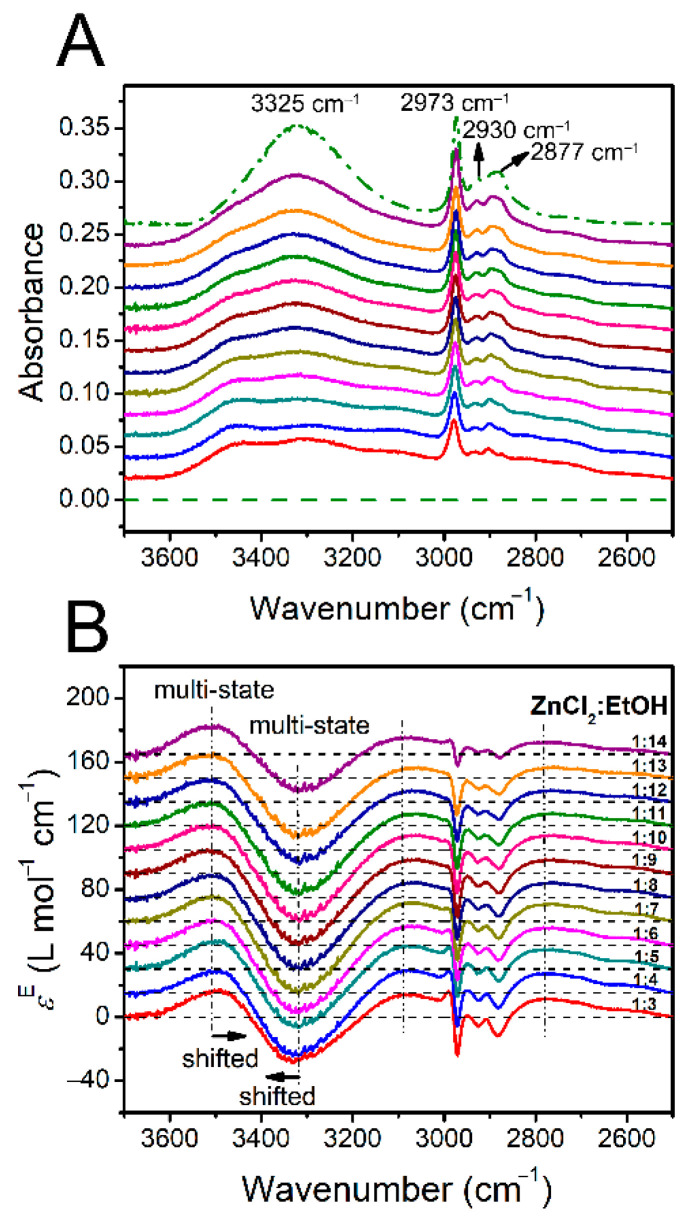
ATR-FTIR (**A**) and excess IR (**B**) spectra of ZnCl_2_-EtOH mixtures in ν(O−H) and ν(C−H) regions. The dash and dash-dotted spectra in (**A**) are pure ZnCl_2_ and EtOH, respectively. The molar ratios of the two components are labeled in (**B**). For a better presentation, the baselines of spectra in (**A**,**B**) have been shifted.

Figure 1A shows the IR absorption spectra of ZnCl_2_-EtOH mixtures with molar ratios between 1:14 and 1:3, equivalent to ~6.7 to 25 mol% ZnCl_2,_ in ν(O−H) and ν(C−H) regions (3700–2500 cm^−1^). The IR spectrum of pure EtOH is also presented as a dash-dotted line. ZnCl_2_ has no absorption in the region. Pure EtOH shows a broad band centered at ~3325 cm^−1^ in the ν(O−H) region (3700–3020 cm^−1^), signaling a wide range of H-bonds in the liquid. In the ν(C−H) region, three bands appear at 2973, 2930 and 2877 cm^−1^, each assigned to at least two vibrational modes [52]. The band at 2973 cm^−1^ is assigned to an overlap of ν_as_(CH_3_) and in-plane ν(CH_2_) of EtOH gauche conformer. The band at 2930 cm^−1^ is assigned to a combination of ν_s_(CH_3_), ν_as_(CH_2_) of EtOH trans conformer and the Fermi resonance of methylene. Finally, the band at 2877 cm^−1^ is an overlap of the Fermi resonance of methyl, ν_s_(CH_2_) of EtOH trans conformer and out-of-plane ν(CH_2_) of EtOH gauche conformer. So, due to the highly overlapped region of ν(C−H), the region will not be considered for further analysis in this study. After the addition of ZnCl_2_, the structures of EtOH are disturbed, leading to changes in the band shapes, positions and intensities. The highest disturbance on the EtOH structures is related to the 1:3 molar ratio mixture (Figure S1). This is accompanied with the emergence of shoulders at both sides of the band center (3325 cm^−1^), suggesting the formation of new species. Here, a species means a molecule or an associate/complex/cluster with a stable structure, as judged with the experimental method. The lowest-wavenumber region (<2800 cm^−1^) of the real spectra (Figure 1A) reveal a low-intensity broad band. The band is assigned to some of the hydroxyls of EtOH molecules, which are largely red-shifted (LRS) when the respective oxygen and hydrogen atoms interact simultaneously with Cl−Zn^+^ and Cl^−^, respectively (see Section 2.2.1). Similar bands have been reported in IR spectra of the aqueous mixtures of two DESs, namely ChCl-xylitol and ChCl-glucose at around 2900 cm^−1^ and were assigned to the formation of strong HO–H**^…^**Cl^−^ H-bonds [61].

Despite the importance of the information obtained from the changes in the positions and intensities of the initial IR bands, further insights are required into the structures and related interactions for a deep spectral analysis. In this regard, we turn to excess absorption spectroscopy, which reveals the concealed changes better than the initial spectra [62,63,64,65,66]. An excess spectrum has one or more positive and negative components, representing the appearing/increasing amount and disappearing/decreasing amount of the involving species in the mixtures, respectively, with respect to their linear prediction upon mixing. The excess spectra in Figure 1B show several positive and negative bands in ν(O−H) and ν(C−H) regions. Due to the highly overlapped bands in ν(C−H), we only focus on ν(O−H). Among the four bands revealed, the negative excess band and the positive one at the highest wavenumbers respectively gradually drift to higher and lower wavenumbers as the concentration of ZnCl_2_ increases. The change in positions of the excess bands implies that the excess bands are made up of more than one excess peak (more than one species). This situation is regarded as the multi-state transformation [63]. The two positive excess bands at the left and right sides of the big negative excess band imply that the hydroxyls in the newly-formed EtOH-containing structures respectively develop weaker and stronger H-bonds in the ZnCl_2_-EtOH mixtures relative to pure EtOH.

### 2.2. Theoretical Investigation on the Interactions between ZnCl_2_ and EtOH

#### 2.2.1. Density Functional Theory

**Figure 2:**
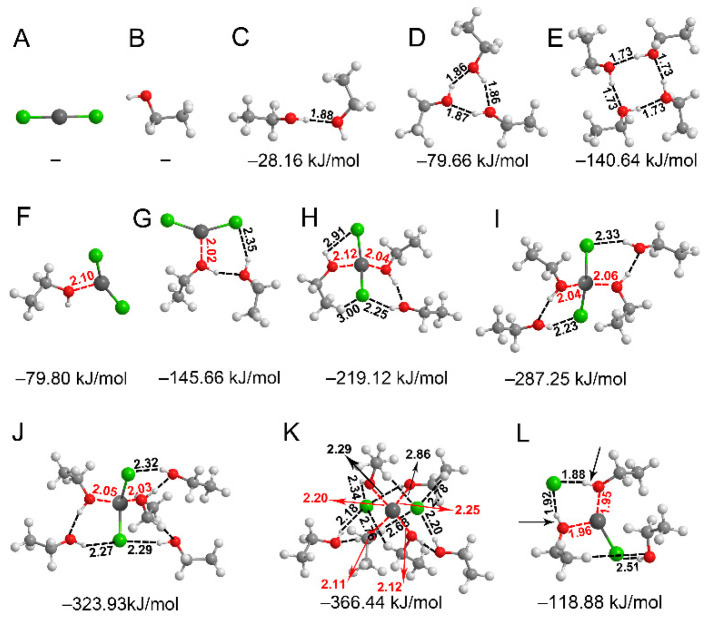
Optimized geometries and interaction energies of ZnCl_2_ (**A**), monomer to tetramer of EtOH (**B**–**E**), ZnCl_2_-*n*EtOH complexes with *n* = 1–6 (**F**–**K**) and a 1:3 molecular ratio complex containing two LRS-OHs marked with black arrows (**L**). The optimization was at the B3LYP-D3/6-311++G(d,p) level of theory. The black and red broken lines respectively indicate the lengths of H-bonds and Zn**←**O coordination bonds/spodium bonds in the unit of Angstrom (Å).

Quantum chemical calculations provide valuable information on the underlying intermolecular interactions and help to interpret the collected spectroscopic data. Here, the structural properties and the intermolecular interactions of ZnCl_2_-*n*EtOH complexes with *n* = 1–6 together with those of pure EtOH structures were explored at B3LYP-D3/6-311++G(d,p) level of theory in gas phase and under a solvent effect. The most stable complexes obtained from gas phase and solvent effect calculations have very close geometries but with different stabilization energies and vibrational frequencies. To judge the formation of H-bonds, the sums of van der Waals atomic radii (Σr_vdW_) of H and O (2.5 Å) and H and Cl (3.0 Å) are used [67]. Besides, the Σr_vdW_ of O and Zn (2.91 Å) [68] is used as the critical value to form Zn**←**O coordination bond or spodium bond. Here, spodium bonds are a new class of noncovalent interactions proposed by Bauzá et al., which are similar to the popular H-bonds and refer to net attractive interactions between Group 12 elements in the periodic table (Zn, Cd, and Hg) and electron-rich atoms [69]. The structures shown in Figure 2 and the consequent discussions are based on the data from gas phase optimizations (other situations are noted). In Figure 2, the H-bonds and coordination bonds are shown by black and red dashed lines, respectively.

Figure 2A shows the structure of ZnCl_2_ which is linear in the absence of any other interacting species. The monomer (compound B), dimer (complex C), cyclic trimer (complex D) and cyclic tetramer (complex E) of EtOH are also shown in Figure 2. These self-associates of EtOH molecules were found as the most stable ones compared to other geometries. The interaction energy per EtOH molecule at B3LYP-D3/311++G(d,p) level of theory increases from −14.08 to −26.55, then to −35.16 kJ/mol for the dimer, the trimer and the tetramer, respectively. These data, together with the shortening of the H-bond length from dimer to tetramer, indicate the cooperativity of the H-bonding interactions in the EtOH clusters. The optimized structures of the ZnCl_2_-EtOH complexes are given in Figure 2F–K. In these complexes, ZnCl_2_ molecules exhibit strong binding interactions with EtOH molecules. That is, Cl atoms form H-bonds with hydrogens of the hydroxyls and Zn atoms, working as Lewis acids, develop coordination bonds with the oxygens of EtOH molecules. Depending on the complexes, the coordination numbers of the Zn atoms are 3, 4 and 6. The formation of coordination bonds of Zn**←**O type reduces the Cl–Zn–Cl angle from 180° in ZnCl_2_ (compound A) to 155°, 146°, 135°, 128° and 127° in complexes F–J, corresponding to 1:1 to 1:5 molecular ratios of complexes. The deformation of the salt structure from a linear geometry (Figure 2A) upon complexation with EtOH is consistent with the previous results on ZnCl_2_, BeCl_2_ or MgCl_2_ molecules interacting with different Lewis bases [70,71,72,73]. Interestingly, the average of the two Zn–Cl bond lengths in each complex increases from 2.10 Å in ZnCl_2_ (compound A) to 2.15, 2.17, 2.22, 2.24 and 2.25 Å in complexes F–J, corresponding to 1:1 to 1:5 molecular ratios of complexes. This is in agreement with the continuous decrease of the electron density at a bond critical point (ρ_BCP_) of the Zn–Cl bonds (0.198, 0.178, 0.171, 0.154, 0.148, 0.145) for compound A and complexes F-J. Moreover, the ρ_BCP_ related to Zn**←**O coordination bonds continuously increases for complexes F–J (0.059, 0.071, 0.125, 0.134, 0.138). For the 1:6 complex (complex K), the Zn**^…^**Cl lengths (2.34 and 2.60 Å) are far beyond the sum of the covalent radii of Zn and Cl (2.24 Å) [74]. This is confirmed by the abrupt drop in the ρ_BCP_ at the center of Zn and Cl (0.098) and the increase of the ρ_BCP_ related to Zn**←**O coordination bonds (0.209) of the 1:6 complex (complex K). Furthermore, the 1:6 complex is the only one with several EtOH molecules developing C–H**^…^**Cl^−^ H-bonds, owing to the higher negative charge on Cl. Some of these results are consistent with those from literatures on the interactions between BeCl_2_ [70,71,72,75] or MgCl_2_ [72] and different Lewis bases. For example, it has been found that the complexes between BeCl_2_ or MgCl_2_ and Lewis bases such as NH_3_ are stabilized through formation of the Be**←**N beryllium bond and Mg**←**N magnesium bond [70,71,72]. This results in the geometrical distortions of the Lewis acids along with the Be-Cl and Mg-Cl bond lengthening. It was also found that among the 1:1 to 1:6 molecular ratios of ZnCl_2_-EtOH complexes (complexes F–K), the |ΔΔE| values, defined as |ΔΔE_1:n,1:n−1_| = |ΔE_1:n_ − ΔE_1:n−1_|, were the greatest for ΔΔE_1:3,1:2_ (73.46 kJ/mol), compared to 65.86 (ΔΔE_1:2,1:1_), 68.13 (ΔΔE_1:4,1:3_), 36.68 (ΔΔE_1:5,1:4_) and 42.51 (ΔΔE_1:6,1:5_) kJ/mol. This implies that ZnCl_2_-3EtOH complex (complex H) is special among the listed complexes and could be a super-stable structure.

Unexpectedly, we found that in some ZnCl_2_-EtOH complexes where ZnCl_2_ dissociates into Cl^−^ and ZnCl^+^, some hydroxyls locate between the charged species, forming the interactive array in the form of [Cl–Zn]^+^**←**O–H**^…^**Cl^−^. On one side, oxygen develops a strong coordination bond with the positively charged zinc, leading to a charge depletion on oxygen and O–H bond weakening. On the other side, the strong H**^…^**Cl^−^ H-bond further lengthens the respective hydroxyl. Consequently, ν(O−H) becomes largely red-shifted. For example, complex L (Figure 2) involves two of such hydroxyls (shown by black arrows) with ν(O−H) of 2874 and 2678 cm^−1^, markedly less than the normal values of ν(O−H). The low-wavenumber broad band appeared at ~2770 cm^−1^ in Figure 1 is suggested to be from such LRS-OHs.

#### 2.2.2. Hirshfeld Charge Analysis

**Figure 3:**
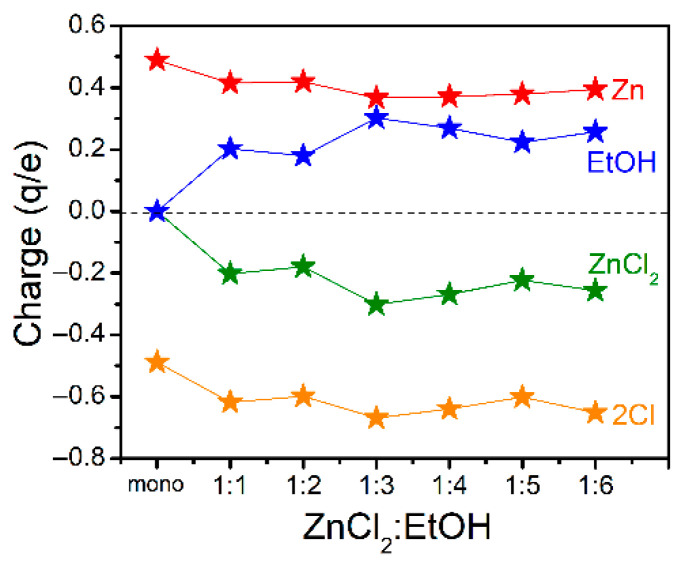
Hirshfeld charges (q/e) on selected atoms and molecules of ZnCl_2_ and EtOH and their complexes. In the figure, mono means monomer (ZnCl_2_ or EtOH).

The Hirshfeld charges [76,77,78] on the atoms and molecules in the ZnCl_2_-*n*EtOH complexes (*n* = 1−6, complexes F–K in Figure 2) together with those of pure ZnCl_2_ and EtOH were examined at the B3LYP-D3/6-311++G(d,p) level of theory. The results are presented in Figure 3 and Table S1. Figure 3 clearly shows that in all the 1:1 to 1:6 complexes, due to the interplays between the salt and the alcohol molecule(s), the salt is negatively charged while the alcohol molecules are positively charged. Here, the charge transfer can be understood as a consequence of the formation of Zn**←**O coordination bonds, though molecular orbital interactions between the involved molecules and even solvent effect of surrounding molecules all play their roles. A similar phenomenon has been reported previously for ZnCl_2_, BeCl_2_ and MgCl_2_, as Lewis acids to interact with Lewis bases through which non-negligible charge transfers from the Lewis bases to the Lewis acid centers [70,71,72,73]. This gives a covalent character to the spodium/beryllium/magnesium bond (Zn/Be/Mg**←**Lewis bases) along with distortions of the Lewis acid units. Interestingly, the received charge by ZnCl_2_ in this study mostly went to the Cls. Each Cl atom in pure ZnCl_2_ has a charge of −0.245 e (Table S1), suggesting the partially ionic character of the Zn–Cl bonds. However, insertion of EtOH molecules increases the negative charge on each Cl atom from −0.309 e in 1:1 complex to −0.326 e in 1:6 complex (Table S1). The Zn–Cl lengths also increase from 2.15 Å (1:1) to 2.47 Å (1:6). These all would finally lead to the dissociation of the Zn–Cl bonds as seen in complexes K and L (Figure 2). In comparison, it has already been reported that the charge transfer through σ^*^_Be-Cl_**←**Lewis base interaction leads to the Be–Cl bond lengthening and dissociation [70,71]. A close inspection of the figure shows that from 1:2 to 1:3 molecular ratios, the charge transfer increment from EtOH(s) to ZnCl_2_ is higher compared to other ratios. Moreover, it was previously seen that the |ΔΔE_1:3,1:2_| value is the greatest among others to represent the super-stable structure of the ZnCl_2_-3EtOH complex. Lastly, since the 1:3 molar ratio mixture has the lowest concentration-normalized absorbance, it has been deduced that ZnCl_2_ has the highest destructive effect on the structures of EtOH in 1:3 molar ratio compared to other mixtures (Figure S1). These all imply the peculiar behavior of the ZnCl_2_-EtOH mixture in a 1:3 molar ratio, suggesting the mixture would be a potential candidate for a DES.

### 2.3. Assignments of the Experimental Excess Peaks

**Figure 4:**
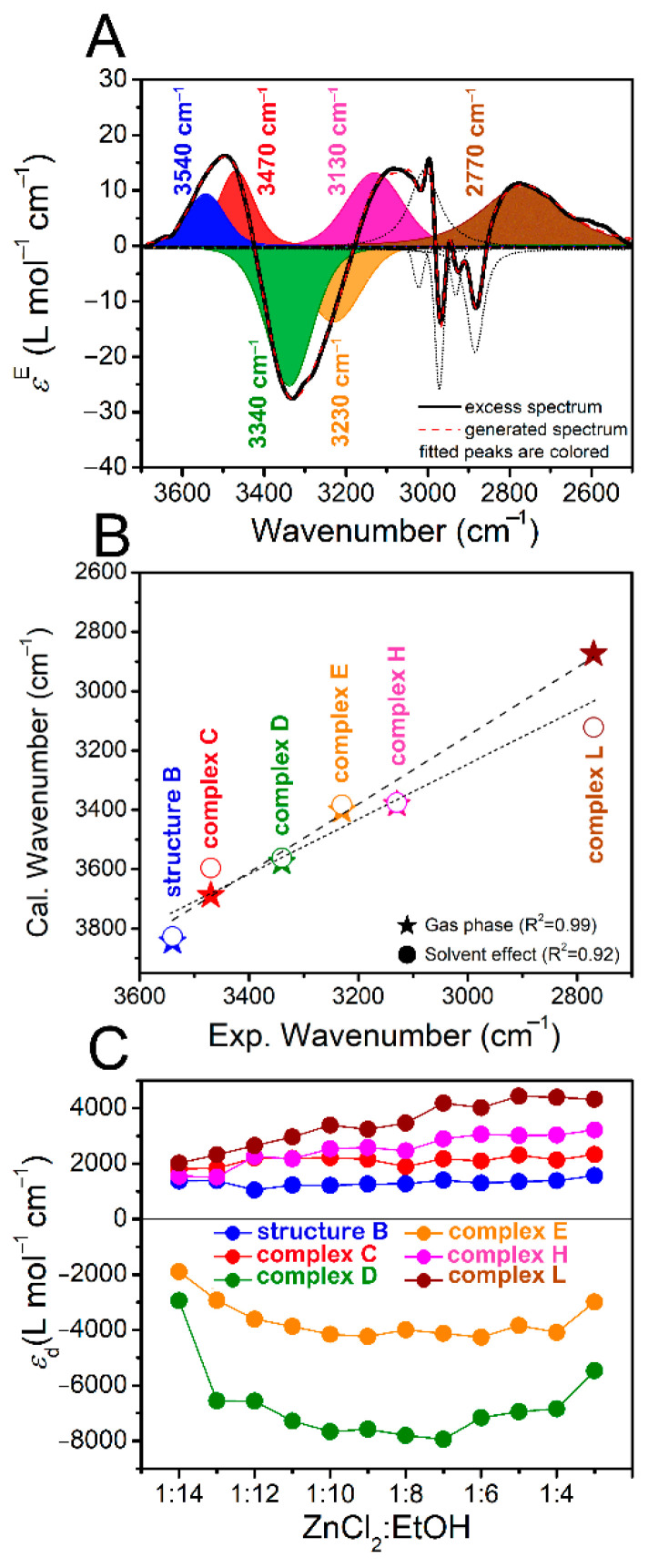
Deconvolution results of the excess spectrum of the ZnCl_2_-EtOH mixture in a 1:3 molar ratio (**A**), relationship between the experimental wavenumbers from the deconvoluted excess peaks and the selected theoretical results at B3LYP-D3 optimized in gas phase or under solvent effect (**B**), molar absorptivity deviation of the hydroxyl-containing deconvoluted excess peaks (**C**). Only the hydroxyl-related deconvoluted peaks are colored in (**A**).

The above discussions on the absorption spectra of ZnCl_2_-EtOH mixtures together with the quantum chemical results helped us to begin a deeper analysis of the data. To this aim, deconvolution procedure was initially performed on the excess bands in ν(O−H) and ν(C−H) regions (Figure 1B). The positive and negative excess components were basically the main guidance in deconvoluting the bands. However, as mentioned earlier, the bands in ν(C−H) region are highly overlapped. So, the band assignments in the region is not considered. According to Figure 1B, out of the four excess bands relating to ν(O−H), the negative excess band and the positive one at the highest wavenumbers are drifting and the other two are fixed. In this regard, each of the two position-fixed excess bands was deconvoluted to a single peak, while each of the position-drifting excess bands were deconvoluted to two peaks to best fit the spectra. Each deconvoluted peak represent one EtOH-containing species. As an example, Figure 4A shows the deconvolution results of the ZnCl_2_-EtOH excess spectrum in a 1:3 molar ratio. For the rest of the excess spectra, similar deconvolutions were carried out (Figure S2). Therefore, the excess spectra are appropriately expressed by four positive peaks at 3540, 3470, 3130 and 2770 cm^−1^ and two negative peaks at 3340 and 3230 cm^−1^.

For the peak attributions, quantum chemical calculations appeared highly beneficial. The structures of the complexes shown in Figure 2 were refined by incorporating solvent effect, taking ethanol as the solvent. Then stretching vibrational frequencies of O-H in these structures were calculated and the results are summarized in supporting information (Table S2). Listed together are those without considering the solvent effect. Depending on the complex, the solvent effect causes a limited red shift or blue shift by different values. In the case of complex L, the hydroxyls have tolerated the largest shift among other complexes when introducing the solvent effect into the calculations. Then, the theoretical wavenumbers of some selected complexes are plotted against experimental ones in Figure 4B. As can be seen, the correlation coefficients with and without taking into account the solvent effect are sufficiently high (R^2^ = 0.99 and 0.92) to confirm the acceptability of the assignments. This method of assignment was previously presented by our group [79]. Accordingly, the peak assignments are as follows: the two negative excess peaks at 3340 and 3230 cm^−1^ are assigned to pure EtOH represented by EtOH trimer (complex D in Figure 2) and EtOH tetramer (complex E). The ZnCl_2_ mixing results in dissociation of the larger clusters of EtOH to smaller ones together with the formation of ZnCl_2_-EtOH complexes. So, the two positive excess peaks at 3540 and 3470 cm^−1^ are ascribed to the appearing EtOH monomer (compound B) and EtOH dimer (complex C). The other positive excess peak at 3130 cm^−1^ is attributed to the ZnCl_2_-3EtOH complex (complex H). Finally, the positive excess peak at 2770 cm^−1^ is assigned to the LRS-OHs in ZnCl_2_-3EtOH complex (complex L). According to the literature studying the vapor pressure of the ZnCl_2_-EtOH [25] and CuCl_2_-EtOH mixtures [80], as the molality of the mixtures increases, the vapor pressure declines. However, the authors have not proposed any reasonable explanation behind this observation. According to the present data, the reduced vapor pressure of ZnCl_2_-EtOH mixtures upon salt mixing can be due to the formation of the strong ZnCl_2_-EtOH complexes such as complexes H and L which were attributed to the excess peaks at 3130 and 2770 cm^−1^, respectively. Similar reasoning might be correct for the CuCl_2_-EtOH mixtures. It needs noting that in the linear correlations (Figure 4B), the calculated wavenumbers, except for complex L, are all intensity-weighted averages of the theoretical O–H absorption positions of the related complexes following the literature method [81]. The quantity variations of the identified species are assessed using the deviation parameter $\varepsilon_{d}$. The results are shown in Figure 4C. The positive and negative data points imply, respectively, the formation or increasing amount and dissociation or decreasing amount of the respective species. As can be seen in the figure, the addition of ZnCl_2_ increasingly dissociates EtOH trimer (complex D) and EtOH tetramer (complex E) up to around the 1:7 molar ratio, after which the dissociation rate of the two complexes decreases. Meanwhile, the ZnCl_2_-3EtOH complex (complex H) and the complex containing LRS-OHs (complex L) increase constantly. Comparatively, the EtOH monomer (compound B) and EtOH dimer (complex C) are quantitatively much less variant under the effect of the salt addition.

### 2.4. Two-Dimensional Correlation Spectroscopy

**Figure 5:**
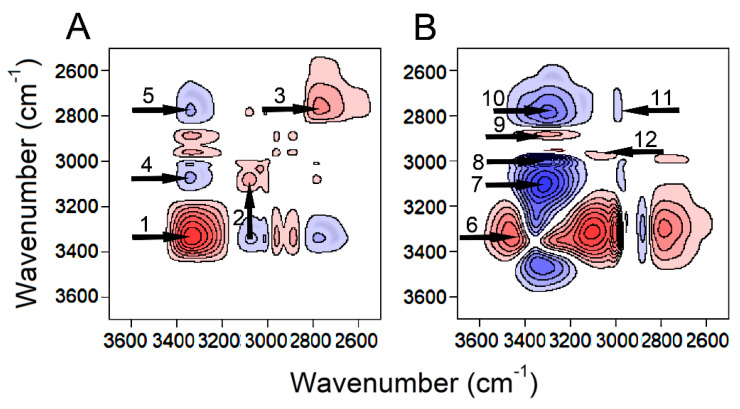
Synchronous (**A**) and asynchronous (**B**) 2D-correlation spectra contour maps of ν(O–H) and ν(C–H) in the process of increasing concentration of ZnCl_2_. Red and blue maps represent positive and negative correlation intensities, respectively.

To further expose the concealed information in the initial IR spectra (Figure 1A) and to partly support the undertaken deconvolution results (Figure 4A), the 2D-COS approach was performed in ν(O−H) and ν(C−H) regions. Initially, all the IR spectra were divided by the concentration of EtOH to obtain normalized spectra. The variations in the absorbance of the normalized spectra are only caused by changes in the strength of the interactions and not the concentration. The synchronous and asynchronous contour maps are shown in Figure 5A,B, respectively. The synchronous contour map reveals three autopeaks associated with ν(O−H) (arrows 1–3). The first autopeak at (3340, 3340) cm^−1^ (arrow 1) corresponds to the negative excess peak attributed to EtOH trimer (complex D in Figure 2). The autopeaks related to EtOH monomer (compound B), EtOH dimer (complex C) and EtOH tetramer (complex E) may have been overshadowed by the prominent autopeak at (3340, 3340) cm^−1^. The second autopeak at (3088, 3088) cm^−1^ (arrow 2) corresponds to the positive excess peak ascribed to the emerging ZnCl_2_-3EtOH complex (complex H). The third autopeak at (2770, 2770) cm^−1^ (arrow 3) corresponds to the LRS-OHs in ZnCl_2_-3EtOH complex (complex L). Moreover, two negative cross peaks at (3340, 3070) cm^−1^ (arrow 4) and (3340, 2770) cm^−1^ (arrow 5) are seen in the synchronous contour map, representing the opposite changing direction of ν(O−H) intensities. Here, the peak at 3340 cm^−1^ is from the disappearing species, while those at 3070 and 2770 cm^−1^ are from the appearing species (Figure S1). The asynchronous contour map discloses several cross peaks at ~ (3480, 3358) cm^−1^, (3317, 3080) cm^−1^, (3317, 3000) cm^−1^, (3317, 2880) cm^−1^, (3280, 2770) cm^−1^, (3000, 2770) cm^−1^ and (3080, 3000) cm^−1^ which are numbered from 6–12, respectively. The wavenumbers at 3480, 3358, 3317, 3280, 3080 and 2770 cm^−1^ are in agreement with the deconvoluted excess peaks (Figure 4A) with R^2^_(excess-2D-COS)_ = 0.97.

## 3. Materials and Methods

### 3.1. Materials and Sample Preparation

Ethanol (EtOH, C_2_H_5_OH, >99%) and zinc chloride (ZnCl_2_, >99.9%) were purchased from Beijing Chemical Plant (Beijing, China) and used without further purification. Different compositions of ZnCl_2_-EtOH mixtures were prepared by weighing for ATR-FTIR analysis. The molar ratios of ZnCl_2_:EtOH are from 1:14 to 1:3, corresponding to ZnCl_2_ concentrations from ~6.7 to 25 mol%.

### 3.2. ATR-FTIR Spectroscopy

ATR-FTIR spectra were acquired using a Nicolet 5700 FTIR spectrometer (Madison, WI, USA) in mid-IR range (4000–650 cm^−1^) at around 25 °C. A mercury-cadmium-telluride (MCT) detector was employed. The ATR was made of Ge crystal with an incident angel of 60° and 7 reflections. The collected spectra were the average of 32 parallel scans with a resolution of 2 cm^−1^ and a zero filling factor of 2. The refractive indices of the solutions were measured with an Abbe refractometer (Shanghai Precision & Scientific Instrument Co., Ltd. Shanghai, China) at ~25 °C. The formula provided by Hansen was considered to determine the light penetration depth and to carry out the ATR corrections.

### 3.3. Excess IR Spectroscopy

For each IR spectrum, excess spectroscopy analysis was performed using the method as described elsewhere [10,82,83]. Briefly, excess absorption is defined as the residual between the absorption of a mixture and that of an ideal mixture:

(1) $$\varepsilon^{E}=\varepsilon -\varepsilon^{\mathrm{ideal}}$$

where $\varepsilon^{E}$ and $\varepsilon^{\mathrm{ideal}}$ are the absorption coefficients of the real mixture and ideal mixture, respectively. The absorption coefficient of the real solution ε is expressed as:

(2) $$\varepsilon =A/d\left(C_{1}+C_{2}\right)$$

where A is the absorbance of the mixture, d is the light path length, $C_{1}$ and $C_{2}$ are molarities of the two components. The absorption coefficient of an ideal mixture is the linear summation of the absorption coefficients of the two components:

(3) $$\varepsilon^{\mathrm{ideal}}=x_{1}\varepsilon_{1}^{*}+x_{2}\varepsilon_{2}^{*}$$

where $x_{1}$ and $x_{2}$ are mole fractions of components 1 and 2, and $\varepsilon_{1}^{*}$ and $\varepsilon_{2}^{*}$ are the molar absorption coefficients of the two components in their pure states, respectively.

The excess molar absorptivity is calculated after inserting Equations (2) and (3) into Equation (1):

(4) $$\varepsilon^{E}=\frac{A}{d\left(C_{1}+C_{2}\right)}-\left(x_{1}\varepsilon_{1}^{*}+x_{2}\varepsilon_{2}^{*}\right)$$

In a binary mixture, $A=A_{1}+A_{2}$, $x_{1}\left(C_{1}+C_{2}\right)=C_{1}$, and $x_{2}\left(C_{1}+C_{2}\right)=C_{2}$. Accordingly, Equation (4) can be expressed as:

(5) $$\varepsilon^{E}=x_{1}\left(\frac{A_{1}}{dC_{1}}-\varepsilon_{1}^{*}\right)+x_{2}\left(\frac{A_{2}}{dC_{2}}-\varepsilon_{2}^{*}\right)=x_{1}\left(\varepsilon_{1}-\varepsilon_{1}^{*}\right)+x_{2}\left(\varepsilon_{2}-\varepsilon_{2}^{*}\right).$$

If a specific band is only from component 1, i.e., EtOH in this work, $x_{2}\left(\varepsilon_{2}-\varepsilon_{2}^{*}\right)=0$. Subsequently, the following equation is acquired to evaluate the deviation of $\varepsilon_{1}$ in the binary mixtures from its pure state [11]. In this work, it is referred to as a deviation parameter.

(6) $$\varepsilon_{d}=\varepsilon_{1}-\varepsilon_{1}^{*}=\frac{\varepsilon^{E}}{x_{1}}$$

### 3.4. Two-Dimensional Correlation Spectroscopy

Two-dimensional correlation spectroscopy (2D-COS) based on the algorithm developed by Noda [60] was performed in the ν(O−H) and ν(C−H) spectral regions of EtOH, using the 2D Shige software, version 1.3 (Shigeaki Morita, Kwansei-Gakuin University). In order to remove the concentration impact on the 2D-COS signals, the initial spectra were divided by the molarity of EtOH. This procedure is known as the modified component-normalization method [84].

### 3.5. Quantum Chemical Calculations

The geometrical and vibrational properties as well as the molecular energies of isolated EtOH and ZnCl_2_ molecules and their complexes in various molecular ratios were carried out using Gaussian 09 [85] at B3LYP-D3/6-311++G(d,p) level of theory in gas phase. The method has been widely used to study the intermolecular interactions in alcoholic systems [52,86,87]. The interaction energy (Δ*E*) of each complex was calculated by subtracting the sum of the stabilization energies of the monomers from that of the complex (Δ*E* = *E*_complex_ − (*E*_monomer1_ + *E*_monomer2_ + …). The basis set superposition error (BSSE) correction was carried out by the counterpoise (CP) method to obtain accurate interaction energies [88]. To study the behavior of the structures under the solvent effect, the polarized continuum model (PCM) was performed at the same level of theory [89,90]. The Hirshfeld charges [76] and the calculations on the topological features based on the atoms in molecules (AIM) theory [91] were performed by using the Multiwfn program, version 3.4 [92].

## 4. Conclusions and Remarks

In this work, the structures, intermolecular interactions and the spectral variations in the mixtures containing EtOH and ZnCl_2_ were explored in a wide range of compositions (1:3 to 1:14 in molar ratios) using FTIR spectroscopy and the results were compared with those from quantum chemical calculations. It was clearly shown that the more the salt was added, the more the H-bonding network of EtOH dissociated. The inspection of the excess spectra assisted by the quantum chemical calculations led us to realize that the transformation process was from EtOH trimer and EtOH tetramer to EtOH monomer, EtOH dimer and ZnCl_2_-3EtOH complex. According to the theoretical findings, the two prominent types of intermolecular interactions governing the solvation process of ZnCl_2_ by EtOH are the O–H**^…^**Cl H-bond and Zn**←**O coordination bond. The detailed analysis of the topological properties of Zn–Cl bonds, together with the abrupt increase in the ionic character of the bond upon addition of EtOH molecules, implied that the Zn–Cl bond tends to dissociate in the solutions. In some of the ZnCl_2_-EtOH complexes, there exist OHs locate in the very special array of Cl–Zn^+^**←**O–H**^…^**Cl^−^ and form super-strong H-bonds. These hydroxyls are largely red-shifted and usually vibrate below the wavenumber of ν(C−H). The formation of such strong ZnCl_2_-EtOH complexes has been proposed to account for the reduced vapor pressure of the ZnCl_2_-EtOH mixtures. In addition, the present work on the interactions of O and Zn would be of help to those with an interest in spodium bonds.

The findings provided in this work on the spectral properties and heterogeneity of ZnCl_2_-EtOH mixtures would shed light on the dark aspects of the solvation phenomena, prominent structures and the decisive underlying interactions in electrolyte alcoholic systems.

## Data Availability

Data is contained within the article and Supplementary Materials.

## Supplementary Materials

The following are available online, Figure S1: ATR-FTIR spectra of ZnCl_2_-EtOH mixtures in ν(O−H) and ν(C−H) regions, normalized by the molarity of EtOH. Figure S2: Deconvolution results of the excess spectra in ν(O−H) region for ZnCl_2_-EtOH mixtures in 1:3 to 1:14 molar ratios. Table S1: Hirshfeld charges on selected atoms and molecules. Table S2: The calculated vibrational frequencies of the structures optimized in gas phase or under the solvent effect.

## Sample Availability

Samples of all the compounds are available from the authors.
