# Supplementary material for: The Structures of ZnCl2-Ethanol Mixtures, a Spectroscopic and Quantum Chemical Calculation Study
Source: Molecules. 2021 Apr 25;26(9):2498. doi: 10.3390/molecules26092498 (PMC8123294; doi:10.3390/molecules26092498)
Supplement: Supplementary file 1 [file molecules-26-02498-s001.zip › molecules-1158850 supplementary final.pdf]

molecules

Supplementary Material

The Structures of $ZnCl_2$-Ethanol Mixtures, a Spectroscopic and Quantum Chemical Calculation Study

Payam Kalhor, Yaqian Wang and Zhiwu Yu*

MOE Key Laboratory of Bioorganic Phosphorous Chemistry and Chemical Biology, Department of Chemistry, Tsinghua University, Beijing 100084, China; kalhor.payam@yahoo.com (P.K.); wangyq19@mails.tsinghua.edu.cn (Y.**Q**.W.)

* Correspondence: yuzhw@tsinghua.edu.cn; Tel.: (+86)-10-6279-2492

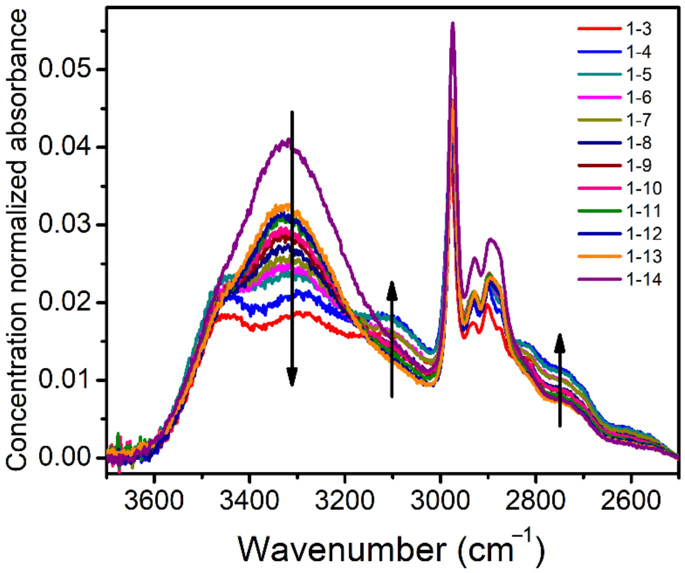

Figure S1. ATR-FTIR spectra of $ZnCl_2$-EtOH mixtures in ν(O–H) and ν(C–H) regions, normalized by the molarity of EtOH.

Table S1. Hirshfeld charges (q/e) on selected atoms and molecules. The data in the parentheses represent the charge changes of the corresponding species upon complexation. Positive and negative values mean decrease and increase in charge, respectively.

Atom/Group	Monomer	ZnCl₂:EtOH Complex					
		1:1	1:2	1:3	1:4	1:5	1:6
$ZnCl_2$	0	−0.202 (−0.202)	−0.180 (−0.180)	−0.302 (−0.302)	−0.269 (−0.269)	−0.223 (−0.223)	−0.257 (−0.257)
Zn	0.489	0.415 (−0.074)	0.419 (−0.070)	0.367 (−0.122)	0.371 (−0.118)	0.379 (−0.110)	0.395 (−0.094)
2Cl	−0.489	−0.618 (−0.129)	−0.599 (−0.110)	−0.669 (−0.180)	−0.640 (−0.151)	−0.602 (−0.113)	−0.652 (−0.163)
EtOH	0	0.202 (0.202)	0.180 (0.180)	0.302 (0.302)	0.269 (0.269)	0.223 (0.223)	0.257 (0.257)
CH_3	0.014	0.040 (0.026)	0.052 (0.038)	0.065 (0.051)	0.083 (0.069)	0.050 (0.036)	0.064 (0.050)

CH_2	0.073	0.136 (0.063)	0.232 (0.159)	0.340 (0.267)	0.419 (0.346)	0.482 (0.409)	0.552 (0.479)
OH	−0.087	−0.027 (0.060)	−0.103 (−0.016)	−0.103 (−0016)	−0.232 (−0.145)	−0.340 (−0.253)	−0.358 (−0.271)

Table S2. The calculated stretching vibrational wavenumbers (cm^{-1}) of O-H in the molecule/complexes shown in Figure 2, optimized in gas phase and under solvent effect. For complexes with more than one hydroxyl (C-K), the intensity-weighed average wavenumbers were used.

Complex	Gas Phase	Solvent Effect
B	3845	3827
C	3688	3596
D	3577	3562
E	3402	3383
F	3807	3818
G	3298	3344
H	3382	3376
I	3353	3362
J	3396	3396
K	3451	3378
L	2874	3254
	2678	3122

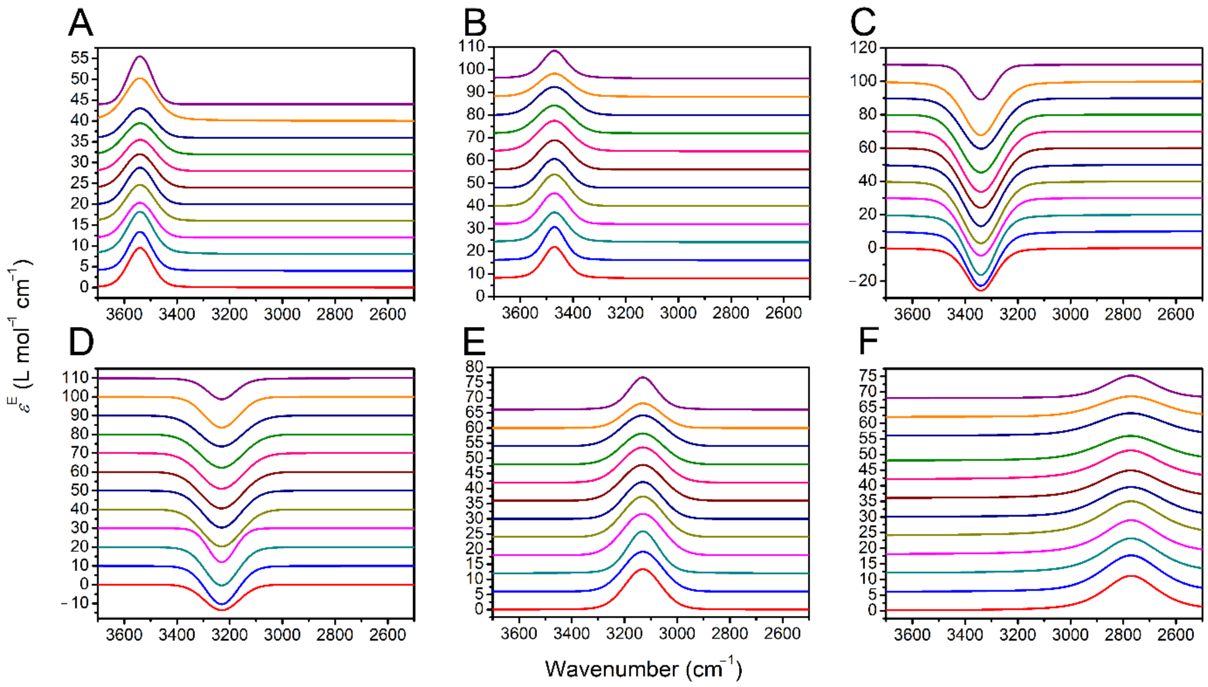

Figure S2. Deconvolution results of the excess spectra in ν(O–H) region for $ZnCl_2$-EtOH mixtures in molar ratios of 1:3, 1:4, 1:5, 1:6, 1:7, 1:8, 1:9, 1:10, 1:11, 1:12, 1:13 and 1:14 from bottom to top. The centers of the deconvoluted peaks are at (**A**) 3540 cm^{-1}, (**B**) 3470 cm^{-1}, (**C**) 3340 cm^{-1}, (**D**) 3230 cm^{-1}, (**E**) 3130 cm^{-1}, and (**F**) 2770 cm^{-1}.
